# Management of an ophthalmology department during COVID-19 pandemic in Milan, Italy

**DOI:** 10.1177/1120672120960334

**Published:** 2020-09-22

**Authors:** Emanuela Filomena Legrottaglie, Laura Balia, Fabrizio Ivo Camesasca, Jose Luis Vallejo-Garcia, Giovanni Fossati, Riccardo Vinciguerra, Pietro Rosetta, Paolo Vinciguerra

**Affiliations:** 1 Humanitas Clinical and Research Center, IRCCS, Rozzano, Milan, Italy; 2 Humanitas San Pio X, Milano, Milan, Italy; 3 Department of Biomedical Sciences, Humanitas University, Pieve Emanuele, Milan, Italy

**Keywords:** COVID-19, lockdown, telephonic triage, keratoconus, corneal cross linking, intravitreal injections

## Abstract

**Purpose:**

Spreading from China, COVID-19 pandemic reached Italy, the first massively involved western nation. At the beginning of March, 2020 in Northern Italy a complete lockdown of activities was imposed. Access to all healthcare providers, was halted for patients with elective problems. We present the management experience of the Humanitas Clinical and Research Center Ophthalmology Department in Rozzano, Milan, Italy, during the lockdown.

**Methods:**

Containment measures were taken to reduce viral transmission and identify infected patients. All planned visits were canceled but for those not deferrable. Social distancing was introduced reducing number of visits per hour. Minor surgery for progressive pathologies was continued. As the lockdown prolonged, we reorganized patient care. All canceled cases were evaluated by electronic medical records analysis and telephonic triage, to identify, recall, and visit patients at risk of vision loss.

**Results:**

From March 9, to April 30, 2020 we performed a total of 930 visits and 612 exams. Some visits (*n* = 698) and exams (*n* = 160) were deemed as necessary for continuity of care and performed as planned. Among the remaining 1283 canceled appointments, after evaluation 144 visits and 32 instrumental exams were classified as urgent and rapidly rescheduled. Performed surgical activities were limited to corneal collagen cross linking (*n* = 39) and intravitreal injections (*n* = 91), compared to 34 and 94, respectively, in the same period of 2019.

**Conclusion:**

In-office activities deemed not deferrable were performed safely. The recall service was highly appreciated by all patients. No patient or staff member reported symptoms of COVID-19.

## Introduction

On December 2019 first cases of pneumonia associated to a new coronavirus appeared in Wuhan, capital of Hubei in China.^1^ Rapidly, its high infectivity and severity characterized this disease as an emergency. The name “COVID-19” (coronavirus disease 2019) was proposed by the World Health Organization (WHO)^2^ but it has also been named “severe acute respiratory syndrome coronavirus 2” (SARS – CoV-2).^3^ The transmission of COVID-19 occurs from person to person through respiratory droplets, direct contacts, and fomites. Airborne aerosol and oral-fecal transmission remain to be confirmed.^4^ Incubation period of the acute severe respiratory infection is usually between 1 and 14 days.^5^ Most commonly, symptoms include fever, dyspnea, and cough; sometimes diarrhea, myalgia, dyspnea, and fatigue; complications such as acute respiratory distress syndrome, arrhythmia, and shock also occur.^6^

COVID-19 transmission involving the eye was hypothesized because the SARS-CoV-2 host receptor ACE 2 has been identified both on cornea and conjunctiva, thus making ocular fluids possible viral carrier.^7,8^ Presence of SARS-Cov-2 virus has been reported in tears or conjunctival sac.^9,10^ Several reports described the possibility of aerosol viral transmission to the conjunctiva when no eye protection was worn.^11–14^ Furthermore, the first physician alerting the world of the new infection was Li Wenliang, an ophthalmologist who probably contracted COVID-19 from an asymptomatic glaucoma patient on February 2020 and succumbed to the disease 1 month later.^15^ The close proximity between ophthalmologist and patient during ophthalmic procedures is a condition fostering viral transmission.

At the beginning of March, facing the severity and diffusion of the disease, Italian government imposed a complete lockdown of activities, initially in Northern Italy and progressively in the whole nation. Hospitals were forced to halt all elective activities, limit access of patients, and dedicate most resources to treat symptomatic COVID-19 patients.

Humanitas Clinical and Research Hospital, located in Rozzano (Milan), is a highly specialized teaching and research hospital. On March 2020 the hospital was identified by Lombardy Regional Government as a regional referral center for COVID-19, oncology, and stroke patients. Our Ophthalmology Department had to rapidly identify which activities should be continued, reduced, or suspended and which measures could be implemented to reduce risk of infection for patients and staff. Hereafter we report our experience during the initial 2 months of the COVID-19 pandemic.

## Methods

The staff of the Ophthalmology Department consists of 17 physicians, five orthoptists, three nurses, and four residents. The Department is located in a small, three-floor building separated from the main hospital structure. Office activity takes place in 18 lanes, 11 for ophthalmological examination, and seven for instrumental exams. Normally, we perform an average of 2680 visits and 1750 exams per month, with ten active subspecialty Services. Minor surgery (i.e. corneal cross-linking, laser refractive surgery, intravitreal injections, and minor adnexal surgery) is usually performed in a small operating room located in the same building. All other surgery is performed in a dedicated, standard operating room located in the main hospital structure.

At the end of February 2020, containment measures were rapidly established to prevent access by potentially infected patients and personnel. Checkpoints were established at every hospital entrance and everybody entering the hospital, staff, and patients, was evaluated for body temperature, symptoms like fever, sore throat, chills, runny nose, and breathing problems. With a temperature higher than 37.5°C, hospital access was denied. Before entering the premises, all persons had to clean hands with alcoholic solution and wear a new surgical mask, provided by the hospital, summarized in Table 1. Reason for hospital access was verified in all patients and, with the exception of disabled, minors, or oncological patients, no accompanying person was allowed. In the Ophthalmology Department healthcare personnel received personal protection equipment (PPE), such as filtering masks KN-95 or PFF2, gloves, protective eyewear, and long-sleeved disposable aprons. Goggles are an efficient tool in reducing the possibility of conjunctival contamination with droplets, mostly during close slit-lamp examination, nevertheless plastic protective shields were also installed on all slit-lamps. “Social distancing” was introduced, reducing the number patients in waiting areas and exam lanes.

**Table 1.** Cleaning and disinfection procedures.

Before entering department	Investigation of COVID19 symptoms and contact with infected (in case of a risk patient, is requested to go home and call their family doctor)
	Supply of surgical mask
	Evaluation of body temperature
	Hands disinfection with ethanol 70% gel
Before entering visit lane	Health care workers’ tasks:
	Environmental sanitation with ethanol 70% or sodium hypochlorite solutions
	Hands hygiene with ethanol 70% concentration gel or chlorhexidine gluconate 4% solution
	Change of nitrile gloves
Medical examination	Patient’s hands disinfection with ethanol 70% concentration gel
	Use of breath shield assembled on slit lamp

On March 8th, the Italian government declared a complete lockdown for the region of Lombardy (DGR 2906, 8th March, 2020). Subsequently, on March 9th all the activities not considered urgent were canceled: the hospital management installed a lockdown of all elective surgery and office activity. It was imperative to protect both patients and healthcare professionals from viral spread. Activity limitations were specified on the law decree n° 3353 emanated on 15th of March, 2020.

At the same time, we considered assistance for acute and chronic sight-threatening conditions as mandatory, according to the DGR 2906 of 8th of March, 2020.

The demand for intensive care beds, respirators, and COVID-19-dedicated wards made mandatory the conversion of several surgical blocks into intensive care units. We thus lost our dedicated standard operating room in the main hospital structure. Elective surgery (i.e. cataracts) and all general anesthesia, urgent or emergency ophthalmological procedures were completely halted and referred to other hospitals, identified as ophthalmic reference hub in Milan.

Booked ophthalmology appointments in the months of March and April, 2020, amounted to 1743 visits and 398 exams. Of these, 698 visits and 160 exams were considered as clinical priority and not canceled. All other 1283 scheduled appointments (1045 visits and 238 instrumental exams) were canceled by phone message, pending re-scheduling. Booking of new visits was likewise stopped.

Minor surgery deemed not deferrable was continued, such as: corneal cross-linking (CXL) and intravitreal injections (IVI). This was possible thanks to the physically separate location of the Ophthalmology Department building from the main structure, where all the COVID-related activity was taking place and to the small operating room located in the Department.

As lockdown continued, we decided to assist our patients remotely, so ophthalmologists reviewed electronic medical records of all canceled appointments, to evaluate urgency of visit or diagnostic exam. Appointments were divided according to subspecialty Services active in our Department. Clinicians contacted by telephone all patients with possible urgent need of care, enquiring about ocular symptoms, vision worsening, problems with sight-preserving therapy, and possible COVID-19 symptoms. Then, according to medical records and referred symptoms, every visit or diagnostic exam was graded with a clinical priority, as defined by National Health System (SSN), RAO (Homogeneous Waiting Groups) criteria as defined by AGENAS (National Agency for Regional Sanitary Services): urgent (U) to be performed within 72 h; less urgent (B), to be performed within 10 days; deferrable (D), performable in a month; programmable (P) performable in a longer period (Table 2).^16^

**Table 2.** Priority grading, SSN (National Health System) RAO classification.

Priority	Scheduling time	Clinical conditions	Symptoms
U	Within 72 h	Ocular trauma, conjunctivitis, keratitis, uveitis	Acute visual loss or scotoma, amaurosis, endophthalmitis, acute glaucoma, new onset anisocoria, ocular inflammations (orbital cellulitis), acute ptosis, foreign body, phosphenes and floaters, acute diplopia, new onset monolateral exophthalmos, chemical and thermal burns, metamorphopsia
B	Within 10 days	Eyelid inflammatory disease, eyelid and orbital tumors	
D	Within 30 days	Glaucoma, diabetic retinopathy, chronic conjunctivitis, evaluation for starting/maintaining systemic therapies (e.g. hydroxychloroquine, corticosteroids, . . .)	
P	Longer period	Long-time loss of vision, pterygium, new diagnosis of diabetes or hypertension, familiarity for glaucoma and others hereditary ocular diseases	
			Rescheduled visits

U: urgent; B: less urgent; D: deferrable; P: programmable.

Patients with a booked visit or exam but no previous medical record were contacted to ascertain reason for the request of examination.

**Figure 1.**
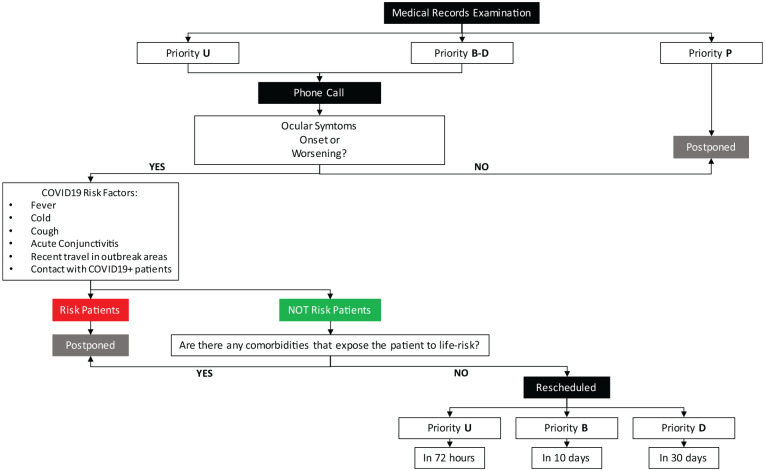
Flowchart summarizing services reorganization protocol based on electronic medical records review and phone calls to patients.

Most of the COVID-19 patients, according to reports from Wuhan, were older men, more likely to have underlying comorbidities such as hypertension, diabetes, cardiovascular disease, and malignancy.^1,17^ Therefore, we decided to postpone visits of patients with two or more comorbidities or, in urgent cases, to evaluate the patient as first in the morning. Patients with U, B, and D priorities were promptly rescheduled, whereas those with P priority were left on hold. A telephonic triage before planning any visit was performed, to exclude fever, flu-like symptoms or recent contact with people with flu-like symptoms. In case of positive findings, patients were canceled and recalled after 14 days. The flow chart of Figure 1 summarizes the reorganization protocol adopted.

## Results

**Figure 2.**
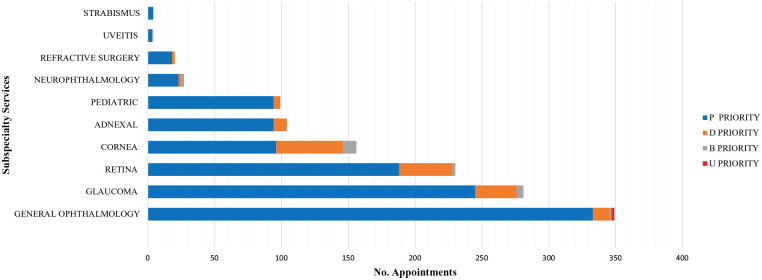
Graph showing the number of booked patients divided according to Subspecialty Services.

Ophthalmologists reviewed all electronic medical records of the 1283 deferred appointments – 1045 visits and 238 exams – contacting by telephone 1043 patients as described in Figure 1. Relatives referred that two patients had succumbed to COVID-19 infection. Two patients were classified as U, 17 as B, 125 as D, and 882 as P priority. Of the 238 booked instrumental exams none was considered as U, 4 were assessed as a B, 28 as a D, and 206 as P priority (Figure 2).

Thus, 144 visits (*n* = 144 patients) and 32 appointments for instrumental exams estimated as U, B, and D priority were rescheduled for rapid execution. Seven patients, properly informed of risk of visual loss when deferring examination, decided nevertheless to postpone it until the end of the lockdown.

**Figure 3.**
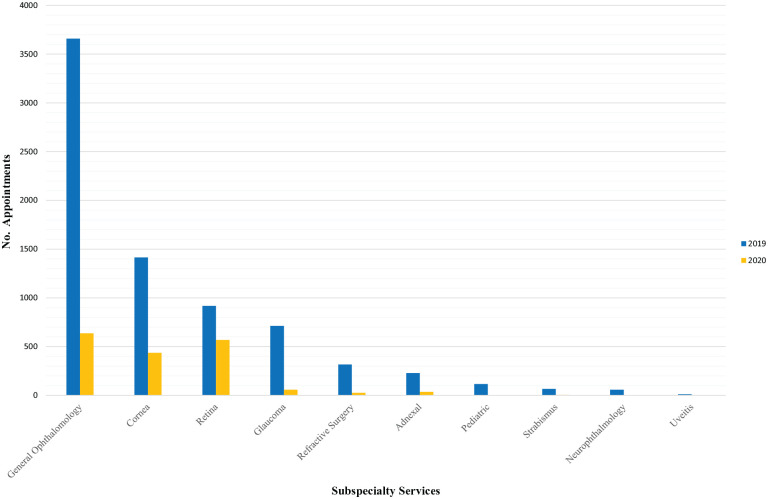
Graph showing the comparison between in-office activities, divided according to Subspecialty Services, during the lockdown period (March 9 - March 30, 2020) and the same period of 2019.

Figure 3 shows a comparison between the lockdown period office activity, divided into subspecialties, with the activity in the same period of 2019. An overall 76.4% decrease in activity was observed, mainly due to reduction in the General Ophthalmology Service.

Table 3 presents priority patients identified and thereafter visited after screening, in General Ophthalmology, Glaucoma, Retina and Cornea Services.

**Table 3.** Priority patients identified, recalled and visited in the General Ophthalmology, Glaucoma, Retina and Cornea Services with related outcomes emerged during evaluations.

General Ophthalmology (*n* = 19)	Glaucoma (*n* = 8)	Retina (*n* = 47)
3 uveitis	2 worsened visual fields	1 wet AMD
1 retinal detachment	2 patients requiring a	2 myopic maculopathies,
4 conjunctivitis	variation of topical therapy	5 retinal ruptures that needed of barrages laser
2 retinal peripheral holes with indication for laser barrage	2 patients requiring iridotomy	1 emovitreous,
		1 pupillary block caused by PDMS (10 years before surgery)
1 iridotomy for low anterior chamber with risk of occlusion	2 significant RNFL reduction on OCT	1 epiretinal membrane with loss of vision
8 posterior vitreous detachments, one of these with hemorrhage		4 patients with ischemic retinal areas who completed argon laser (PRP)
1 exposed ocular prosthesis in an enucleated patient who required surgery		1 corioretinal sierous central treated
		50 PRP and macular grid in 31 diabetic patients
Cornea		
Priority (B, *n* = 10 and D, *n* = 50)	Outcome	
1. Young keratoconic patients not treated with CXL	1a. Young keratoconic patients with significative progression, amenable of CXL in one eye (*n* = 17)	
	1b. Young keratoconic patients without significative progression, to be controlled (*n* = 17)	
2. Keratoconic patients previously treated with CXL with suspect of ectasia relapse	2. Keratoconic patients previously treated with CXL, without progression (*n* = 17)	
3. Patients transplanted in one eye only with controlateral eye disease	3a. Bullous keratopathy in list for DMEK (*n* = 1)	
	3b. Corneal leucoma in list for SCTK (*n* = 1)	
	3c. Adenovirus keratitis (*n* = 2)	
4. Patients with recent onset of astigmatism (young patients with keratoconus familiarity and patients with history of refractive surgery)	4. Patients with recent astigmatism without ectasia (*n* = 5)	

CXL: corneal cross-linking; DMEK: Descemet membrane endothelial keratoplasty; SCTK: sequential customized therapeutic keratectomy.

Among the deferred patients contacted and visited, none subsequently had problems related to COVID-19 or presented at an Emergency Room.

We hereafter describe in detail the actions taken by Cornea and Retina Services. We deemed keratoconus in young patients a dangerous situation passible of irreversible worsening if overlooked. In 47 eyes of 46 patients, corneal cross-linking (CXL) was considered as mandatory even during lockdown. We treated 39 eyes of 38 patients (mean age 25 ± 6.8 SD). Immediate postoperative monitoring was continued. Eight patients preferred to differ treatment. In the same period of 2019, 34 keratoconic eyes were treated with CXL.

Follow-up visits of keratoconic patients that underwent CXL in January and February, 2020, were performed. Transplanted patients with history of rejection, herpes keratitis or recent transplant were also monitored identifying one rejection, two suture hydrolysis, and two herpetic keratitis. All these complications were timely identified, treated, and successfully controlled. In the canceled group of appointments for cornea patients, we identified 10 patients with B priority and 50 with D priority. We considered as priorities untreated young keratoconic patients, as well as patients transplanted in one eye and with affected, untreated contralateral eye. We recalled and visited these patients, identifying 17 young untreated keratoconic patients (mean age, 18 ± 6 years) with progression and indication for CXL (Table 3), to be performed subsequently.

Usually, the main load of our Retina Service is managing patients requiring anti-VEGF intravitreal injections (IVI). We decided to continue treating these patients to avoid the risk of irreversible visual loss. We adopted all possible safety measures to reduce the risk of viral infection in this delicate group of patients. Advanced mean age of these patients appeared as particularly worrysome, given the severity of COVID-19 in senile patients.

We usually adopt a treat-and-extend protocol for anti-VEGF in age-related macular degeneration with subretinal neovascularization. Injections were performed in the small operating room within the Ophthalmology Department building. The macular treatment clinic performs the visit and IVI in a 6-h shift per week. This allotted time was increased to 15 h per week in order to avoid overcrowding. In total, we performed 91 IVI in the period from the 9th of March to the 30th of April; 94 were performed in the same period during 2019. During the appointment call, all special measures introduced by the hospital were explained, however 13 patients decided to skip treatment. Two of these were later known to be hospitalized for COVID-19.

Medical records of canceled patients were reviewed and recalled by retina specialists, defining their priority. Forty-seven patients were identified as with priority and visited, as reported in Table 3.

In summary, we observed a total 76.4% reduction in activity (78.7% visits, 77.3% exams), in particular 71.3% in the Cornea Service (66.0% visits, 73.2% exams) and 44.0% in the Retina Service (36.9% visits, 52.2% exams).

## Discussion

The first reported case of COVID-19 in Italy was on February 21st 2020.^18^ Infection spread rapidly in the Nation, heavily involving the North and particularly the regions of Lombardy, Veneto and Emilia Romagna, with more than 200,000 infected patients and more than 28,000 casualties by the end of April.^18^ Lombardy suffered 56% of the Italian casualties.^18^ In the report produced by SARS-CoV-2 Italian Surveillance Group Italy the mean age of patients dying for COVID-19 was of 79 years, with affected men being 74.2% of patients and 60.7% of the sample with three or more comorbidities.^18^

Close proximity to the patient, particularly during slit lamp examination, characterize ophthalmologists a “high risk category” requiring personal protection devices (PPD) to reduce risk of infection via droplets.^19^

On March 18th, 2020, the American Academy of Ophthalmology (AAO) emanated an alert for Ophthal-mologists with guidelines to reduce risk of COVID-19 transmission. AAO strongly recommended ophthalmologists to provide only urgent care and suggested to keep the waiting room as empty as possible; encouraging use of slit-lamp barriers and surgical masks or better PPD for patients and physicians.^15^ On April 17, 2020 AAO recommended possible reprise of ophthalmological activity according to regional epidemiological situation and exclusively with adequate PPE.

We carefully considered these recommendations and made the changes reported in Table 1 to improve safety, as well as conducting a telephonic triage before any visit, to exclude fever, flu-like symptoms or recent contact with people with flu-like symptoms. We continued clinical activity for visits, exams, and minor surgery deemed necessary for continuity of care and immediate postoperative evaluations. All other scheduled visits, exams or procedures were canceled. When lockdown duration appeared to prolong, we shifted our strategy and worked to identify all suspended patients for possible risk of visual loss, organizing and managing a structured recall and examination procedure.

Our Department is a national referral center for the diagnosis and treatment of keratoconus, the most common corneal ectatic disease and the second most common cause of corneal transplant worldwide. Keratoconus is a disease progressive in time, especially in young people as well as when associated to syndromes such as Down syndrome.^20,21^ The introduction of CXL has reduced the number of patients requiring corneal transplant.^22,23^ Due to the increasing number of CXL procedures in our Department, we have a waiting period of averagely 3 months between the date of CXL indication and the day of surgery. Indications for CXL are progression of keratoconus defined as an increase of Kmax or astigmatism, a subjective loss of vision and/or corneal thinning of more than 20 microns of minimal corneal thickness.^24–27^

During the COVID-19 lockdown the decision to continue CXL surgery appeared controversial, mostly in consideration of the risk of infection. Nevertheless, we considered that what appeared to be an undefined waiting period before lockdown interruption would simply allow keratoconus to progress, especially in young patients. Our decision to perform CXL was based on the experience of Romano and Vinciguerra.^28^ They found a greater incidence of progression in young patients, especially below the age of 18. This was associated to a Kmax increase of more than 1 diopter and irreversible reduction in visual acuity, while waiting for CXL treatment.^28^ According to Chatzis and Hafezi, children under 18 years should undergo CXL as soon as possible.^29^ Reported results suggested that optimal waiting time should be no longer than 12 weeks for patients older than 18 years and less than 6 weeks for younger ones.^28^ In consideration of these data, in our Department young patients represent a priority for CXL and we carefully avoid waiting periods longer than 6 to 8 weeks. For all these reasons, we strived to continue CXL treatments also during lockdown. We didn’t observe any complication in the patients undergoing CXL, nor anyone reported subsequent COVID-19 infection. Calling in young keratoconic patients that were canceled, allowed us to identify progression in 50% of patients, thus performing timely CXL.

Retinal Service patients were scheduled on the basis of age and comorbidity for the death rates in Italy. Patients at higher risk of death if infected, because of sex, age, and comorbidity, were appointed within the first slots of the day. With lockdown, the reduction of our retinal activity was related to the withdrawal of routine or periodical controls of patients without acute or severe problems. Vitreoretinal procedures done before the lockdown were controlled within the first month or until gas tamponade gas absent or below 20% filling. Although some pandemic guidelines recommend diversely, we decided not to delay IVI in patients with macular edema related to diabetes, uveitis or retinal vein occlusion because of their increased risk of irreversible visual loss if left untreated.^30^

Limits of this study are its retrospective nature and the difficulty in comparing results with similar experiences, due to the exceptionality of this pandemic, unprecedented in the past 100 years. We confronted with the need to reduce all unnecessary activities, to define rapidly those not deferrable, then to maintain vision care in patients at risk during a lockdown of uncertain duration. Nevertheless, since we decided to provide the best possible eye care while maintaining low the risk of COVID-19 diffusion, we identified and exploited several key features of our situation:

1. Structural advantage: the Humanitas Ophthalmology Department building is separated from the main hospital structure, thus theoretically it was not contaminated with known COVID-19 patients, suspects or health workers directly involved with COVID-19 care.
2. Closure of most of the ophthalmological activity reduced the everyday appointments workload, allowing appropriate patient separation in waiting areas while attending visits and treatments.
3. For the same reason, more ophthalmologists than usual were available for clinical evaluation, diagnostic exams, and therapeutical choices, often directly administered in the same session, as happened with anti-VEGF IVI.
4. Patient permanence time was reduced to a minimum.

The best possible way to deliver care in such sudden, critical situations still remains uncertain. We report our experience and the rational approach we adopted for clinical and safety decisions based on the conditions available in our center during the pandemic onset.

Due to these clinical and strategical choices, to safety procedures, location, characteristics and size of our facilities, we could maintain CXL and IVI levels practically unchanged with respect to 2019, without inducing any known case of COVID-19 among patients or staff. Contacting all scheduled patients gave them the certainty that their vision was our priority and they were not left alone even during this unprecedented and scaring pandemic.
